# Setting maximum levels for lead in game meat in EC regulations: An adjunct to replacement of lead ammunition

**DOI:** 10.1007/s13280-020-01336-6

**Published:** 2020-05-25

**Authors:** Vernon G. Thomas, Deborah J. Pain, Niels Kanstrup, Rhys E. Green

**Affiliations:** 1 Department of Integrative Biology, College of Biological Science, University of Guelph, Guelph, ON N1G 2W1 Canada; 2 Department of Zoology, University of Cambridge, David Attenborough Building, Pembroke Street, Cambridge, CB2 3QZ UK; 3 Department of Bioscience, Aarhus University, Grenåvej 14, Rønde, 8410 Aarhus, Denmark

**Keywords:** Europe, Game meat, Hunting, International trade, Regulation, Scavengers

## Abstract

Each year, hunters from 12 of the 27 European Union (EU) countries and the UK shoot over 6 million large game mammals, 12 million rabbits and hares and over 80 million birds. They support an international game meat market worth over 1.1 thousand million Euros. Animals shot with lead ammunition frequently contain lead fragments in the carcass which contaminate meals made from game meat with concentrations of lead substantially above the maximum allowable level (ML) set by European Commission Regulation EC1881/2006 for meat from domesticated animals. This poses a health risk to frequent consumers of wild-shot game meat, with children and pregnant women being particularly vulnerable. Total replacement of lead rifle and shotgun ammunition with available non-toxic alternatives is needed for all hunting in EU nations to prevent exposure of humans and wildlife to ammunition-derived lead and to allow the depletion of the long-term environmental legacy of lead from spent ammunition. We propose that EC1881/2006 is amended to incorporate an ML for game meats as a supplementary measure to the replacement of lead ammunition. This would harmonise food safety standards for lead in meats traded across and imported into the EU.

## Introduction

Modern European hunting results in game meat that is consumed either by hunters, their families or associates and enters the retail market place and restaurants (Schulp et al. 2014). The trade in game meat is large (FAO 2018), both within and among European nations, and between Europe and other countries. This trade generates large revenues (Schulp et al. 2014; FAO 2018) that offset the costs of maintaining habitats on shooting estates. Human consumption of wild game meat is increasing, including the UK (BASC 2018, 2019), reflecting a preference for ‘unfarmed’ meat and the promotion of wild game as a healthy alternative to other meats (Taggart et al. 2011). Campaigns to promote game meat consumption are active in the UK (BASC 2019; CA 2019), as is the Danish promotion of game meat in schools (DJA 2019).

Lead ammunition frequently leaves tiny fragments of lead dispersed widely through the meat of both large game shot with bullets (Hunt et al. 2009) and birds and other small game shot with lead gunshot pellets (Pain et al. 2010). This source of lead is biologically available (Green and Pain 2012) and is not easily removed, especially from the flesh of small game animals (Green and Pain 2019). It thus poses a health risk to those who frequently consume game shot with lead ammunition and to children and pregnant women who are especially vulnerable to the effects of lead (Pain et al. 2010; Green and Pain 2012, 2019; Knutsen et al. 2015). There is a large and growing awareness of the effects of ammunition-derived dietary lead on human health and well-being and their associated societal impacts and costs (Delahay and Spray 2015; Kanstrup et al. 2019; Pain et al. 2019a). Non-lead substitutes for lead shotgun and rifle ammunition have been developed and are available to European hunters (Thomas 2015; Thomas et al. 2016), but no European-wide regulation exists to require their use for game hunting (Mateo and Kanstrup 2019).

European Commission Regulation (Council Directive 92/5/EEC) concerns the procurement and handling of game meat (Bertolini et al. 2005), but does not mention the use of lead ammunition in taking wild game. European Commission Regulation EC1881/2006 sets maximum levels (MLs) of lead allowed in traded meats from domesticated bovine animals, sheep, pigs and poultry, but also from less frequently eaten meats from wild animals, including cephalopods and bivalve molluscs. However, no ML has been set for lead in game meat. The European Commission is aware of the elevated lead levels found in game animals (EFSA 2010, 2012), and the food standards or safety agencies of a number of European Union (EU) nations have issued new advice intended to reduce or eliminate health risks associated with the consumption of lead-contaminated game meat. This is intended for frequent consumers and vulnerable pregnant women, women of pregnancy age and children (Knutsen et al. 2015; ANSES 2018; Gerofke et al. 2018, 2019). However, this increase in awareness and the provision of health advice has not resulted in EU or any national regulations concerning lead MLs in game meat.

The present paper supplements the reviews of ECHA (2018), Pain et al. (2019a, b) and Green and Pain (2019) of the effects of lead ammunition use on human and wildlife health, and the analysis of Gerofke et al. (2019) on the sources and consequences of lead in game meat in Germany. We indicate the scale of game hunting and trade in Europe, and the health risks posed by lead from frequent ingestion of wild-shot game meat. We then describe the advantages of amending the European Commission Regulation that sets the ML for lead in domestic meat so that it includes meat from wild game animals. In particular, we argue that this action would complement and facilitate the essential transition to non-lead ammunition for European hunting, which would benefit people, wildlife and domestic animals (Pain et al. 2019a).

## Hunting and trade in game across the European Union

Most game hunting in Europe is conducted on privately owned lands and game meat trade occurs via private agencies. Statistics on the numbers of animals killed each season, by species, and by region are obtained by voluntary questionnaires or statutory reporting (for birds). The Birds Directive 2009/147/EC sets the framework for hunting legislation across the EU. This specifies how, when and where 82 bird species may be hunted legally and requires the provision of data on hunting bags at regular intervals. In terms of voluntary questionnaires, FAO (2018) reported data collected from United Nations Economic Commission for Europe (UNECE) countries using a questionnaire survey in 2016 and 2017. The objective of this FAO pilot study was to improve knowledge and understanding of game meat production and trade. Game was taken to comprise all hunted birds and mammals, such as partridge (*Perdix perdix* and *Alectoris* spp.), pheasant (*Phasianus colchicus*), hare (*Lepus europaeus*), deer including roe deer (*Capreolus capreolus*), red deer (*Cervus* spp.), fallow deer (*Dama dama*) and European elk (*Alces alces*), wild boar (*Sus scrofa*) and chamois (*Rupicapra rupicapra*) that are available for consumption, but the study excluded farmed game (mostly deer and wild boar). The study focussed particularly on game species that use forested or forest associated habitats. Although reporting requirements for birds are mandatory under the Birds Directive, data provided both from this survey and voluntary schemes varied substantially in coverage and quality.

The fresh weight of game killed and its traded value (FAO 2018) are presented in Tables 1 and 2. These figures represent only the most important mammalian and avian game species and came from those countries that replied most fully to the questionnaires. We recommend that FAO (2018) is consulted for information on hunted species of lesser economic importance to the game trade. The data in Tables 1 and 2 are annual means averaged across recent annual reports. The numbers vary from year to year because of variation in wild game recruitment patterns, hunter effort and market economic conditions. The 13 EU countries that replied to the survey on numbers of animals killed have 5 465 000 hunters, representing 82% of the 6 667 770 hunters in the EU 28 in 2010 (FACE 2010). Assuming that a similar number of mammals are killed per hunter by the remaining 18% of hunters gives an estimated annual kill across the EU of 6 282 841 large mammals (3 species of deer plus wild boar) and 12 269 575 brown hares and rabbits.

**Table 1:** Annual numbers of wild mammals shot in 13 EU countries^a,b^ and tonnage of game produced. Data are taken from FAO (2018) and represent the most important game species hunted

Species	Annual kill (number of countries that reported)	Annual tonnage (assumed weight of individual animals in kg)
Roe deer *Capreolus capreolus*	2 294 324 (13)	45 886 (20)
Red deer *Cervus elaphus*	480 464 (12)	72 070 (150)
Fallow deer *Dama dama*	156 032 (12)	9362 (60)
Wild boar *Sus scrofa*	2 218 687 (11)	155 308 (70)
Brown hares *Lepus europaeus*	2 039 436 (11)	7750 (3.8)
Rabbit *Oryctolagus cuniculus*	8 016 884 (7)	16 033 (2)
Total mammal kill	15 205 827	306 409

^a^Croatia, Czech Republic, Finland, France, Germany, Ireland, Italy, Lithuania, Luxembourg, Poland, Spain, Sweden, UK. The 13 countries that replied to the survey have 5 465 000 hunters (82%) of the 6 667 770 in the EU 28 as of 2010 (FACE 2010). Assuming that a similar number of mammals are killed per hunter by the remaining 18% of hunters, this gives an estimated kill of 6 282 841 large mammals and 12 269 575 brown hares and rabbits

^b^The total kill of birds approaches 88 million in the EU, from the data of Hirschfeld et al. (2019) and Green and Pain (2015: for the UK) extrapolated to include all EU countries (see text). Data from FAO (2018) on bird kills were too sparse from many countries to allow reasonable representation

**Table 2:** The annual tonnage and traded values of game meat reported by six EU nations in FAO (2018). These numbers refer to the principal species of mammals and birds involved in the game markets. The values in US$ were converted to Euros using the exchange factor 0.908

Six nations reporting trade data^a^	Traded quantity in tonnes/y		Traded value in million Euros/y	
	Imports	Exports	Imports	Exports
	70 881	127 696	178.22	298.36

^a^Croatia, Finland, Lithuania, Poland, Spain, Sweden

The 6 EU countries that reported trade data have 1 771 000 hunters (26.56%) of the 6 667 770 reported in the EU in 2010 (FACE 2010). Assuming a direct relationship between the numbers of hunters and the level of export trade gives an estimated export trade value in excess of 1123 million Euros a year for the whole of the EU

Data on numbers of birds killed in the EU are sparse in FAO (2018). Hirschfeld et al. (2019) found that almost 52 million birds (51 808) were reported as shot annually in the EU, but these data excluded the UK, Greece, Ireland and the Netherlands, where 20% of shooters are reported to live (FACE 2010). In the UK, Green and Pain (2015) used available data to make a conservative estimate of 28.1 million birds shot annually, although these data are from a decade ago and numbers shot are likely to have increased, along with increases in numbers of released gamebirds (primarily pheasants and red-legged partridges *Alectoris rufa*). Adding the UK figure to that of Hirschfeld et al. (2019) gives a total of c.80 million birds shot in the EU, but excluding Greece, Ireland and the Netherlands. These latter three countries contain 9.2% of the total number of hunters in the EU (FACE 2010). If we assume that a similar average number of birds are shot per hunter in these countries, this suggests that about 88 million birds are shot per year. This is not dissimilar to the totals given in the FAO (2018) voluntary questionnaire. FAO data showed that 12 EU countries with 4 665 000 hunters (in 2010: FACE 2010) reported shooting 38 766 554 birds of selected species. To this we can add UK figures of 800 000 hunters shooting 28.1 million birds (FACE 2010; Green and Pain 2015) giving a total of 5 465 000 million hunters (82% of total hunters) shooting 66 866 554 birds. Extrapolating this to the total number of EU hunters in 2010: 6 667 770 (FACE 2010) gives a total of 81 544 000 birds hunted. This may be an underestimate given that not all species were reported and numbers have increased in the UK, but is broadly similar to the estimate of Hirschfeld et al. (2019) for the EU.

Despite the reporting limitations inherent in the FAO (2018) survey, the results indicate a large annual kill of mammals (Table 1) and birds as indicated above. Fewer countries reported trade data. Data in Table 2 are based on the principal mammal and bird species traded, which are deer and boar, waterfowl, pheasant and other non-wetland gamebirds. The annual traded values of the EU imports and exports are large (Table 2; FAO 2018). The 6 EU countries that reported trade data have 1 771 000 hunters (26.56%) of the 6 667 770 reported in the EU in 2010 (FACE 2010). By assuming a direct relationship between the numbers of hunters and the level of export trade, extrapolation of the 298 363 005 Euros reported by those 6 countries (Table 2) gives an estimated export trade value in excess of 1123 million Euros a year for the whole of the EU. This is unlikely to be precise as there may not be a direct relationship between the number of hunters and the level of trade, but this gives a broad idea of the overall value of trade in the most important species.

## Health problems posed by lead fragments from ammunition in game meat

**Fig. 1:**
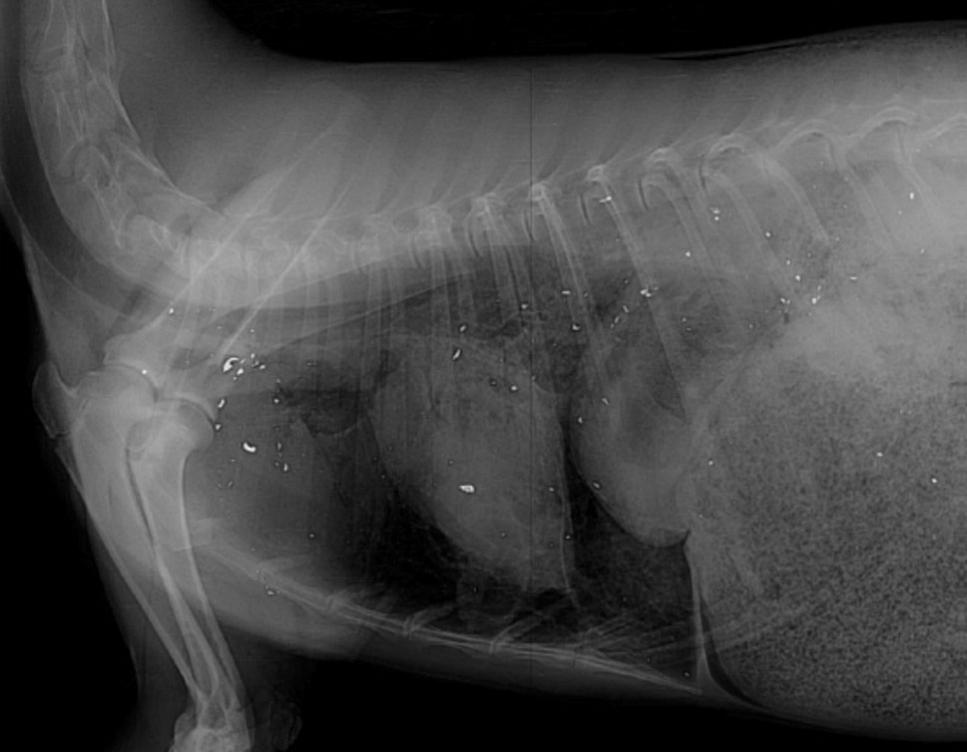
Radiograph of a roe deer shot with a single unbonded lead rifle bullet, showing the extent of the bullet’s fragmentation and the distance of fragments’ spread from the entry site. Most of the small fragments would not likely be removed prior to butchering and retail sale, thereby exposing the consumer. Photo credit, Oliver Krone, Leibniz Institute for Zoo and Wildlife Research, Berlin, Germany

**Fig. 2:**
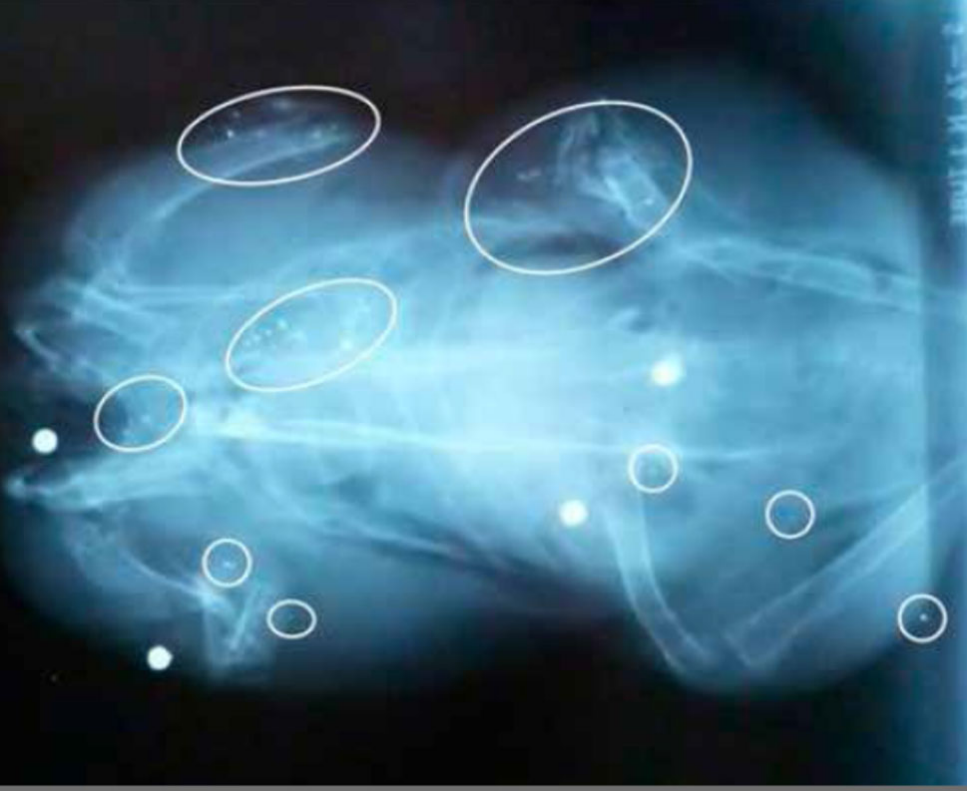
X-ray of a woodpigeon illustrating four gunshot and numerous small radio-dense fragments. Radio-dense fragments may trace the passage of shot through the bird; some fragments are close to bone suggesting fragmentation on impact, others are not. Reproduced from Fig. 1 of Pain et al. (2010)

Lead hunting bullets are designed to expand on entering an animal, and many small lead fragments can be released from the bullet’s core (Fig. 1). The extent of fragmentation depends on the type of bullet, its terminal velocity and the tissues penetrated, especially bone (Dobrowolska and Melosik 2008; Trinogga et al. 2019). Unbonded jacketed lead bullets fragment more than costlier bonded jacketed bullets. While it is common practice for hunters and game handlers to remove flesh around the point of bullet’s entry, small distant fragments are likely to evade removal and, ultimately, be consumed by humans. Non-lead rifle bullets are designed not to fragment, thus avoiding contamination of the carcass. Copper, which has very low toxicity compared to lead, is frequently used for non-lead bullets, and research has indicted that this does not present a health risk (Krone et al. 2019). Lead gunshot often remains in birds until prepared for cooking, or even after cooking. Multiple shot may be found in both the vital and the non-vital parts of the body, including small fragments produced when pellets strike hard tissues (Fig. 2). While intact shot are visible, many are not removed prior to cooking, which could increase the solubilisation and availability of lead to humans (Mateo et al. 2007).

Removal of lead shot and bullet fragments is impractical in small game animals like gamebirds (Green and Pain 2019) and results in discarding of a considerable quantity of meat in large game animals. In Norway, discarding meat close to wound channels results in approximately 200 tonnes of contaminated meat being discarded annually, representing a loss of around 3 million Euros (Kanstrup et al. 2018). The experimental removal of whole shot and large fragments of lead gunshot to simulate what consumers would do at the table still results in lead levels in meat that are, on average, more than an order of magnitude higher than the EC MLs set for the meat of domestic animals (Pain et al. 2010; Lindboe et al. 2012). Many waterfowl ingest spent lead shot whose lead is absorbed and deposited in the organs (primarily liver and kidney) and the skeleton. Other birds may carry throughout life lead shot embedded in tissues from prior hunting encounters (Pain et al. 2019b). Even though such birds may be killed later by hunters using non-lead shot, these birds may enter markets with lead levels exceeding current EC MLs for meat and offal, especially in the livers and kidneys (Guitart et al. 2002). The only pragmatic solution to this problem is the appropriate labelling of retailed waterfowl carcasses that alert consumers to a potential health risk from lead. In large mammals killed with lead-based rifle bullets, the lead contamination may vary considerably throughout the carcass. Animals killed with a single heart–lung shot may have bullet fragments widely dispersed through thoracic meat (e.g. Hunt et al. 2009; Fig. 1), but meat from the hind quarters may be lead-free (Gerofke et al. 2018). Mincing the meat from the thoracic region would homogenise the lead within the retailed product (Lindboe et al. 2012; Vogt and Tysnes 2015).

This issue is not unique to Europe and arises wherever hunters use lead ammunition (Pain and Green 2019; Thomas et al. 2019). The health risk to humans increases with the annual consumption of contaminated game meat (Taggart et al. 2011; Green and Pain 2012, 2015), the type of game eaten (e.g. mammals vs. birds), and with the vulnerability of the consumer to the effects of dietary lead (especially children and pregnant women).

## The human and societal cost of lead exposure from game meat consumption

While absorbed lead affects most body systems in humans, critical effects were considered by the Panel on Contaminants in the Food Chain (CONTAM Panel) of the European Food Safety Authority (EFSA) to be developmental neurotoxicity in young children and cardiovascular effects and nephrotoxicity in adults (EFSA 2010). Children and foetuses are particularly sensitive to dietary exposure and are considered to be the most vulnerable group. This is both because they absorb a higher proportion of the lead ingested, and because children’s developing brains are especially susceptible to the effects of chronic lead exposure, even when blood lead concentrations indicate a low level of exposure (Lanphear et al. 2005; Budtz-Jørgensen 2010; EFSA 2010).

Pain et al. (2019a) estimated the economic costs of reduced IQ in those children deemed at risk from ingestion of lead from ammunition in the diet. Such a calculation requires an estimate of the numbers of children exposed to sufficient dietary lead from ammunition to result in blood lead levels associated with reduced IQ. A 1 point (1%) reduction in IQ was considered significant at a population level by EFSA (2010). In the UK, it has been estimated that 4000–48 000 children were at risk from incurring a one point or more reduction in IQ as a result of their level of exposure to dietary lead from game meat (Green and Pain 2015). Another survey in the UK by the British Association for Shooting and Conservation and the Countryside Alliance (BASC/CA) found that, in the UK shooting community alone, 9000 (midpoint of 5500–12 500) young (8 years or younger) children consume at least one game meal per week averaged over the year (reported in LAG 2014). As this level of consumption generally exceeds the amount of dietary lead exposure associated with a 1 point reduction in IQ (Green and Pain 2012, 2015), it seems probable that at least 10 000 children in the UK are at risk. Pain et al. (2019a) assumed that the ratio of children at risk in the UK relative to the number of UK hunters would be similar across the EU. This gave an estimate of 83 000 or more children across the EU27 who may be at risk of an IQ reduction of 1 point.

The societal costs of reduced IQ have been estimated in various ways by different authors and relate to impacts on academic achievement and/or decreased productivity in later life (e.g. Schwartz 1994; Grosse et al. 2002; ECHA 2011; Bierkens et al. 2012; Monahan et al. 2015). Using the range of values from the last three of these studies, Pain et al. (2019a) estimated that the consumption of lead shot game by the cohort of children 8 years old or younger within the EU was linked to a potential loss in IQ worth €322 million to €830 million. This equates to an annualised (i.e. ongoing and cumulative) cost to society of €40 million–€104 million for every year that lead-contaminated game continues to be consumed at current levels. The authors considered that the actual cost may be higher than estimated because some children will be exposed to more lead from game than is associated with a 1 point reduced IQ, with greater concomitant risks, and also because some studies indicate that in some EU countries, more people may be ‘high-level’ consumers of game, relative to the national number of hunters, than in the UK (see Pain et al. 2019a).

We are unaware of other attempts to monetise the possible health effects associated with elevated blood lead from consumption of lead shot game. Increased blood lead levels are associated with increased risk of cardiovascular disease and of chronic kidney disease (EFSA 2010) and may contribute to antisocial behaviour and increased crime rates (e.g. Campbell et al. 2018; Sampson and Winter 2018), with related costs to both the individuals concerned and society in general. Based on a 2008 survey on blood lead concentrations in French children aged one to 6 years old, Pichery et al. (2011) estimated the monetary benefits in terms of avoided national costs if threshold values for lead toxicity above 15 μg/L, 24 μg/L and 100 μg/L were introduced, at €22.72 thousand million, €10.72 thousand million and €0.44 thousand million, respectively. It is notable that more people appear to eat game frequently and be ‘high-level’ consumers than might previously have been supposed. Green and Pain (2019), by extrapolating from UK surveys and reviewing studies from elsewhere, estimated this to be approximately 5 million people (1% of the population) in the EU. In some EU countries, this has been estimated to be several times higher (e.g. 3% in Italy: Ferri et al. 2017).

## Impacts of lead ammunition ingestion on scavengers

Hunters customarily discard the organs and entrails of killed animals in the field. These entrails frequently contain lead bullet fragments, and the gut piles are often eaten by avian and mammalian scavengers (Stokke et al. 2017; Hampton et al. 2018). At least 5–6 million gut piles from deer and boars may be discarded annually throughout Europe (based on Table 1) and pose a lead exposure risk to scavengers. Whole animals shot by hunters may be left in the field, either deliberately as pests, or accidentally, when not retrieved. Waterfowl hunting, for example, is often accompanied by large unintentional crippling losses when birds are hit but not retrieved (Falk et al. 2006). These carcasses are eventually fed on by scavengers which may then ingest the shot or bullet fragments. These sources of lead exposure are additional to those from discarded gut piles.

The toxic effects of dietary lead on scavenging species are well documented (Golden et al. 2016; Krone 2018). Pain et al. (2019b) indicated that many species of scavenging and predatory raptors (Old and New World vultures, eagles, hawks, falcons, and owls) are susceptible to this form of lead exposure. Toxic effects in raptors range from overt mortality to abnormal behaviour (Ecke et al. 2017; Pain et al. 2019b). This form of lead exposure occurs globally and probably affects every European scavenging raptorial species (Krone 2018; Pain et al. 2019b). Exposure to ammunition-derived lead is a threat to at least nine species of raptor globally classified as threatened or near threatened with extinction (Krone 2018; Pain et al. 2019b). Apex predatory mammals such as bears (*Ursus* spp.) also scavenge the remains of large game animal kills and so may also be at risk (Legagneux et al. 2014). The voluntary use of non-lead rifle ammunition in some parts of the USA has been related to reductions in lead exposure and ingestion by raptors (Kelly et al. 2011). A similar change would probably have beneficial effects were it introduced in Europe. Preventing lead exposure and toxicosis in scavenging species has been the main justification for passing federal laws requiring the use of non-lead shot for hunting waterfowl throughout the USA (1991) and Canada (1999) (Thomas et al. 2019). In 2019, California became the first state jurisdiction to require non-lead hunting shotgun and rifle ammunition for all types of hunting throughout the state, mainly to prevent lead exposure of several raptorial species (Thomas et al. 2019). Any regulation of lead use intended to protect human health would have a simultaneous and positive effect on the health of all scavenging species, especially raptors.

## Potential effects of an amendment of European Commission regulations dealing with lead in meat

Although exposure of humans to elevated levels of dietary lead derived from ammunition has been known for decades, this exposure pathway is absent from the Alimentarius Code of Practice on reducing exposure to lead in food (Codex Alimentarius 2004) and no ML for lead in human foodstuffs derived from wild-shot game animals is set in the Codex Alimentarius General Standard for Contaminants and Toxins (Codex Alimentarius 2018). It is difficult to understand why the ammunition route of exposure to dietary lead has not been mentioned within Codex Alimentarius and why MLs have not been set for game, given that levels of exposure in frequent consumers of game meat shot with lead ammunition are high.

This important exposure route needs to be acknowledged (Taggart et al. 2011) and health-protective measures put in place. Taggart et al. (2011) noted the large discrepancy between what is legally considered to be safe in terms of lead content of European foods and what is actually present in wild game meats. EC Regulation 1881/2006 does not set MLs of lead in game meats (EC 2006). This may have been because the committees setting these levels assumed (1) that lead projectiles would remain intact, and therefore present little risk to consumers who would remove projectiles from food at the table and/or (2) that relatively few people eat wild game frequently. Recent research has shown that neither of these assumptions is correct. Firstly, because lead bullets and gunshot pellets often fragment on impact leaving behind tiny lead particles, their removal is not practical in small game animals like gamebirds (Green and Pain 2019). In large game animals like deer, shot with bullets, removal of contaminated tissue results in considerable meat wastage. After removal of large visible lead fragments in gamebirds prior to cooking, lead levels in the meat were still on average, more than an order of magnitude above the EU MLs set for the muscle of domestic livestock and poultry (Pain et al. 2010). Even meals made from gamebirds with no visible lead pellets or large fragments in the carcass often had lead concentrations considerably higher than the MLs set for other meats. Secondly, food standards generally aim to protect specific consumer groups as well as the general public. Many who frequently consume wild game are likely to be sport and subsistence hunters and their families and friends. In some countries, such as the UK and Denmark, game animals, especially gamebirds, are often given to employees of game shoots and consumed by them and their families. This represents a form of occupational exposure to lead, which, while strictly regulated in other contexts, is not in the case of game shooting. Some people may consume game for health reasons and it is widely promoted as such in the UK. Although many recipes for game are given in websites and literature promoting the consumption of game, most do not include information on removing lead-contaminated tissues. Green and Pain (2019) suggested that the numbers of people who frequently consume wild game are higher than previously assumed, perhaps about 1% of the population of the EU (c. 5 million people). Those choosing to eat game for ethical or health reasons could purchase it from retailers where a lead ML could be applied.

It might be thought that testing game meat for lead would be difficult because lead from ammunition is unevenly distributed across the tissues of wild-shot animals, so that multiple samples would need to be analysed for comparison with the ML. Additionally, if large lead fragments were present, the lead levels would be misleadingly high. However, protocols are readily available in which large particles of ammunition are removed prior to analysis to simulate culinary practices (Pain et al. 2010).

The relevant MLs of lead of concern in European Commission Regulation (EC) 1881/2006, Setting Maximum Levels of Certain Contaminants in Foodstuffs, Annex, Section 3, Metals, Lead, are as follows:

Section 3.1.3. Meat (excluding offal) of bovine animals, sheep, pigs and poultry (0.10 mg/kg).

Section 3.1.4. Offal of bovine animals, sheep, pigs and poultry (0.50 mg/kg) (EC 2006).

We consider below the effects of amending these Sections to:

Section 3.1.3. Meat (excluding offal) of bovine animals, sheep, pigs, poultry and wild game mammals and birds (0.10 mg/kg).

Section 3.1.4. Offal of bovine animals, sheep, pigs, poultry and wild game mammals and birds (0.50 mg/kg).

This amendment would harmonise the regulations across all domestically reared and wild game animals within the EU. It would, if passed, apply to all EU nations and other countries across which wild game meat and meat products are traded commercially. Establishing an EC ML for lead in traded game meat would require means to both monitor and enforce the regulation. We propose that the same monitoring and lead testing procedures used for domestically reared meat could be applied to commercial wild game. The consumers of game meat obtained from retail outlets, such as restaurants, shops and supermarkets, would be affected by the lead content of the portions served or bought, rather than the lead content of the entire carcass. This would have implications for the scale of monitoring and testing of the meat from large game animals, but for gamebirds, the lead content of the whole animal bought or served is usually the issue.

## Discussion

The exclusion of wild game from European Commission lead regulations is paradoxical given the large annual kill of game in Europe and its associated markets. The proposed amendment to harmonise lead regulations for game meat with domesticated meat would, if enacted, reduce human lead exposure from marketed game. Simultaneously, lead ingestion by scavengers would be reduced by hunters’ use of non-lead ammunition.

The use of lead ammunition is now recognised as unsustainable (Kanstrup et al. 2018). The transition to use of non-lead shotgun and rifle ammunition is not hampered by the availability of lead substitutes (Thomas 2015; Thomas et al. 2016; Kanstrup and Thomas 2019), their effectiveness (Kanstrup et al. 2016; Stokke et al. 2019) or their cost (Thomas 2015; Kanstrup and Thomas 2019). Availability of both types of ammunition is dependent upon demand, which, in turn, depends upon legislation regulating the ammunition types that may be used for hunting (Thomas 2015). In some countries, the increased human consumption of wild game reflects a preference by some for ‘unfarmed’ meat. This provides an opportunity for the hunting community to promote the strategy of supplying society with natural products. Setting a ML for lead in game would enhance both food safety and the sustainability of hunting.

The transition to non-toxic shot in Europe is occurring slowly and has been driven largely by concerns about lead exposure to wetland bird species which ingest spent lead shot. Lead shot use is restricted legally in 23 European countries, not all of which are EU Member States (Mateo and Kanstrup 2019). The extent of the restriction varies. In Denmark, it is illegal to possess lead shot cartridges, so all hunters and target shooters use non-lead shot. The Netherlands also bans use of lead shot for hunting and shooting. Many nations, including those banning lead shot use over wetlands, still allow lead shot to be used for non-wetland game hunting. Legislation requiring the use of non-lead rifle bullets has not been passed at the national level in any European country, and only Germany requires such ammunition to be used in several regions (Mateo and Kanstrup 2019). Regulations also restrict the use of lead ammunition in at least an additional 10 countries beyond Europe (Stroud 2015; Mateo and Kanstrup 2019), including the USA and Canada, and the use of all types of lead ammunition for hunting has been banned throughout California State (AB 711 2013).

An EU-wide restriction on the use of lead gunshot for shooting in and over wetlands was proposed by the European Chemicals Agency under REACH^1^ at the request of the European Commission (ECHA 2018; SEAC 2018), primarily to protect waterbirds and harmonise measures taken across the EU. An ECHA Annex XV Investigation Report (ECHA/PR/18/14 2018) contended that further measures could be considered, extending the restriction to all shooting, to protect both human health and predatory and scavenging birds. At the request of the Commission, ECHA is now preparing a broader restriction proposal on the placing on the market and use of lead in ammunition used in both wetlands and other terrains (ECHA 2019).

In their Investigation Report (ECHA 2018), ECHA concluded that “the most effective manner to deal with lead is at the source, i.e. through a regulatory action on the use of lead ammunition. Other measures (setting maximum lead levels in game meat) are protective for human health, but would not be protective enough for scavengers and raptors. Additionally, such a limit value would not protect hunters that consume their own meat.” While agreeing with most of these conclusions, we contend that setting MLs is needed in addition to the replacement of lead ammunition and that these measures are complementary. A ban on the use of lead ammunition would provide a harmonised level of protection to raptors and scavengers and would remove ammunition-derived lead from the meat of wild-shot game animals traded freely within the EU’s single market. However, a ban on the use of lead ammunition alone would not harmonise lead safety standards in traded domestic and game meats within the EU, nor deal with the issue of game meat that is imported into the EU. The setting of MLs for lead in game within Regulation 1881/2006 would achieve both, and additionally provide some level of health-protective compliance monitoring, were a ban on lead ammunition implemented. Achieving this goal would also alert other global jurisdictions about the need for health-protective international food safety standards.

The risks from exposure to elevated dietary lead are global, affecting subsistence communities in some of the most remote regions on earth, such as the Peruvian Amazon (Cartró-Sabaté et al. 2019), sport shooting communities in the EU and across the world, and urban consumers who purchase wild game. We therefore encourage the Joint FAO/WHO Expert Committee on Food Additives (JECFA) to include this issue on its subsequent agendas.

While international regulation requiring the replacement of lead ammunition with non-toxic alternatives is urgently needed, it is not yet in place. Should the setting of MLs precede such a ban, it would simultaneously reduce exposure of wild birds to lead ammunition. However, the setting of MLs, while in our view desirable, would not alone be sufficiently protective to wildlife and might not protect the majority of people at risk who frequently consume game. Hunters could continue using lead ammunition to kill animals for their personal consumption, thereby exposing them and their families to lead remnants in the game meat. While Table 1 indicates the numbers of animals killed annually, it does not reveal the numbers consumed only by hunters and their families. However, it is assumed that the majority of ‘high level’ or frequent consumers of game are hunters, their families and associates as illustrated by studies from the UK (LAG 2014; Green and Pain 2015) and other countries (e.g. in Italy, Ferri et al. 2017). In the UK, where game is commonly sold in supermarkets and other retail outlets, game sales have been reported to be increasing year on year for the last 5 years to 2018, with a 5% increase in 2018 (BASC 2019) as a result of game meat promotion campaigns. Nonetheless, it remains widely assumed that across the EU the majority of game consumed in the country of origin is consumed locally by hunters and their associates. However, this obviously does not apply to traded game meat.

Despite a lack of national and international regulation setting standards for lead in game meat, there have been recent examples of trade-initiated voluntary restrictions on lead ammunition. Forest Enterprise England (FE—an executive agency of The Forestry Commission, a UK Government Department) requires their staff to use non-lead ammunition for deer and boar culling from 2016. This decision resulted from evidence that lead from lead ammunition contaminates carcasses and that FE’s marketing position could be seriously damaged if they continued to put lead-contaminated meat into the human food chain when proven alternatives exist. Forest Enterprise Scotland is also transitioning to lead-free ammunition to shoot deer and feral pigs.^2^ Together, these forestry agencies put over 900 tonnes of venison into the human food chain annually. In 2019, the UK supermarket Waitrose, the largest national retailer of game meat, indicated that, as of the 2020/2021 season, it would sell only game meat that was killed with non-lead ammunition (Barkham 2019; Waitrose 2019). Other UK supermarkets have also indicated that they will act similarly.

## Conclusions

The risks arising from the use of lead ammunition are incurred by wild animals, humans and the environment, and there is a great need to replace lead ammunition with non-toxic alternatives. The lead contamination of game meat is an important issue in Europe because game meat is both eaten locally and traded globally. Setting MLs of lead in harmony with EC regulations on lead in meat and offal from domesticated animals is critical to complement the regulated use of lead-free ammunition and protect all people in the EU who purchase and regularly consume game meat. This change can be achieved by an amendment of existing regulations on the EC MLs of lead in meat. An EC action on MLs would also stimulate setting international standards applicable to game meats imported into the EU. MLs would also provide a monitoring mechanism for Member States to measure compliance with eventual bans on the use of lead ammunition. Substitutes for all types of lead ammunition are available and in use in various European jurisdictions and pose no economic barrier to their use. Current initiatives of the EC on lead reduction from ammunition are highly appropriate. If realised, they portend benefits to the health of humans and wildlife species that ingest lead (Mateo et al. 2014), and the soils and waters of the environment that receive so much discharged lead each year.

## Footnotes

1 The EU’s Regulation, Evaluation, Authorisation and Restriction of Chemicals.

2 https://markavery.info/2017/12/06/forest-enterprise-nontoxic-ammunition/. https://markavery.info/2018/12/20/lead-free-venison-from-scotland/.
